# Editorial: Development of next generation bio stimulants for sustainable agriculture

**DOI:** 10.3389/fpls.2024.1383749

**Published:** 2024-04-08

**Authors:** Sruti Bajpai, Pushp Sheel Shukla, Balakrishnan Prithiviraj, Alan T. Critchley, Nagarajan Nivetha

**Affiliations:** ^1^ Marine Bio-Products Research Laboratory, Department of Plant, Food and Environmental Sciences, Dalhousie University, Truro, NS, Canada; ^2^ Research and Development Division, Sea6 Energy Private Limited, Centre for Cellular and Molecular Platforms, National Centre for Biological Sciences-Tata Institute of Fundamental Research, Bengaluru, India; ^3^ Verschuren Centre for Sustainability in Energy and Environment, Cape Breton, NS, Canada

**Keywords:** Next-generation biostimulants, sustainable agriculture, nutrient-use-efficiency, plant growth, stress tolerance

Chemical fertilizers have been at the core of the agricultural production system in the last century, but their excessive usage poses a threat to the ecosystem (Del Buono, 2021; Shukla et al.). Additionally, only 18-49% of the applied fertilizer was used by the plants, and the remaining is lost to runoff to aquatic bodies causing eutrophication and leaching (Gomiero et al., 2011). In the current scenario, there is an urgent requirement to develop a sustainable agricultural system to address fundamental issues related to economical agricultural production in an ecologically friendly manner (Tahat et al., 2020). Plant biostimulants are gaining interest as an alternative sustainable strategy to improve the innate ability of treated plants to cope with stress tolerance and efficiently utilize the available nutrients (Shukla et al.; Nephali et al., 2020; Shukla and Prithiviraj, 2021). These biostimulants were derived from seaweeds, microbes, and other natural sources (Shukla et al.). There is a demonstrable need to develop more potent biostimulants and explore their additional functionalities so that agriculture can be more resilient and sustainable. This involves the exploration of synergistic actions by the combination of different classes of biostimulants, identification of new sources with higher bioactivity, development of novel extraction methods, and understanding the new functionalities for the existing biostimulant products (Rouphael and Colla, 2018; Aeron et al., 2021; Johnson et al., 2023). This editorial summarizes the contributions of different researchers toward the development of next-generation biostimulants for sustainable agriculture.

Spinelli et al. demonstrated the biostimulant activity of culture filtrate obtained from the fungus, *Chaetomium globosum* and *Minimedusa polyspora* on the leaf area, fresh and dry biomass, and root:shoot ratio of *Cichorium intybus.* The bioactive compounds present in the culture filtrate of *C. globosum* induced the biosynthesis of phenylalanine and chicoric acid in the roots of *C. intybus*. In contrast, the culture filtrate of *M. polyspora* induced 4-OH-benzaote in the roots of *C. intybus*. Trehalose, chitosan, humic acids, and gamma-aminobutyric acid were screened for their biostimulant activity on the germination and growth of maize (Li et al.). Interestingly, these biostimulants increased the nitrogen, potassium, phosphorous content and grain quality of maize. Even though they did not have any effect on germination rate, the seedlings from humic acid treatments had significant drought resistance. Humic acid effectively improves the synthesis of unsaturated fatty acids, alkaloids and metabolites involved in improving abiotic stress tolerance in those treated seedlings. The publications by Spinelli et al. and Li et al. provided evidence that selected bioactive molecules could elicit specific plant growth responses.

Seaweeds (macroalgae) are a major source of marine bioactive molecules which are widely reported to induce growth, stress tolerance, and nutrient-use-efficiencies in treated plants. There are some differences between species of seaweeds used to make the extract, as well as specificities of foliar or soil applications, dose rate and frequencies to elicit specific plant responses (Shukla et al., 2016, 2019; Shukla and Prithiviraj, 2021; Deolu-Ajayi et al., 2022; Trivedi et al., 2023). Seaweeds belonging to the Rhodophyceae (i.e., red algae) had previously been less explored as a source of biostimulants for plants than those belonging to the Phaeophyceae (brown seaweeds). Various types of extracts of *Kappaphycus alvarezii*, a cultivated, tropical red seaweed, have been recently explored for their applications as plant biostimulants (Trivedi et al., 2023). Most of these reports reviewed focused on concentrated “sap” extracted from the algal thallus by processes such as crushing or mincing (Ghosh et al., 2015; Vaghela et al., 2022). The sap from *K. alvarezii* reportedly contains phenols, flavonoids, steroids, quinones, carbohydrates, protein, lipids, carotenoids and ascorbic acid (Vaghela et al., 2022). Shukla et al. reported that LBS6, a commercial differentiated product derived from *K alvarezii* thalli, by mixing the sap together with chemically hydrolyzed pulp demonstrated the induced expansion of cucumber cotyledons by regulating the expression of genes involved in cell division, expansion and proliferation. In addition, this specific *K. alvarezii-*derived biostimulant, also regulated the expression of genes involved in the endogenous phytohormone regulation of the treated plants, primarily playing an important role in cell division, expansion, and proliferation (Shukla et al.). These same authors also translated the beneficial effects of *K. alvarezii*-derived biostimulant (LBS6) in whole plant assay too, where those plants sprayed with the product showed better growth in terms of leaf area, fresh and dry biomass. The treated plants demonstrated modulation of electron and proton transport-related pathways which help better growth by efficiently utilizing the photosynthetically available radiation (Shukla et al.). LBS6, when applied as a root drench to *Pisum sativum* grown under optimum, excessive, and deficient nitrogen (N) conditions, improved the growth and plastochron under optimum and N-deficient conditions (Shukla et al.). LBS6-treated plants showed a reduction in N deficiency-induced lipid peroxidation and improved photosynthetic parameters. In *P. sativum*, LBS6 was shown to regulate the differential expression of the genes involved with N uptake, transport, assimilation, and remobilization (Shukla et al.).

Morales-Sierra et al. screened extracts from the red algae: *Bonnemaisonia hamifera, Galaxaura rugosa, Dasycladus vermicularis*, the green alga: *Ulva clathrata*, and the brown seaweeds: *Cystoseira foeniculacea, C. humilis, Lobophora dagamae, Colpomenia sinuosa* and *Halopteris scoparia* all for their various biostimulant activities when applied to tomato seedlings. Their results showed that an extract from the red alga, *Galaxaura rugosa* exhibited the highest biostimulant activity in tomato seedlings when grown under water deficit stress. *G. rugosa-*derived extract mitigated water-deficit-stress by improving both CO_2_ fixation and water-use-efficiency (Morales-Sierra et al.). The beneficial effects of the *G. rugosa-*derived extract in conferring water-deficit-stress tolerance was attributed to the induced expression of abscisic acid-responsive genes (Morales-Sierra et al.). Taken together the three publications of Morales-Sierra et al., Shukla et al., and Shukla et al. provided missing evidence regarding the potential of specific red seaweeds as a source of biostimulant extracts for sustainable agriculture. In another study, Vaghela et al. evaluated the beneficial effect of minimally processed aqueous homogenates (MPHs) derived from the red *K. alvarezii* and the brown seaweed *Sargassum wightii* on the growth of *Zea mays.* These MPHs were rich in bioactive compounds for plant growth such as retronecine, tyrosyl-glycine, hexyl 2-furoate, 1-phosphatidyl-1D-myo-inositol, 12-(2,3-dihydroycyclopentyl)-2-dodecanone, and trihomomethionin. The above publications collectively provide much needed, new insights to the efficacy and biological activities of various seaweed-based biostimulants, as well and insights to their specific modes of action in improving plant growth, stress tolerance, and nutrient-use-efficiency. It is patently obvious from the above that not all extracts of seaweeds (SWEs) are the same. Different extracts, different biotic and abiotic responses of treated plants. There are considerable differences based on both raw materials and extraction/hydrolysis processes. Understanding these not insignificant differences is essential to establishing product value in the market, as well as new and future applications for sustainable agriculture.

Protein hydrolysates (PHs) are produced by the chemical or enzymatic hydrolysis of the proteins extracted from different animal or plant bio-products of agro-industrial wastes (Colla et al., 2015). PHs serve as plant biostimulants and are known to regulate key molecular, biochemical, and physiological processes involved in treated plant growth and stress tolerance (Colla et al., 2014). Rouphael et al. showed the effects of vegetal protein hydrolysates (VPH), free copper and copper-complexed peptides, and amino acids of vegetal origin (Cu and Cu-VPH), and their combination on the growth of *Ocimum basilicum.* Specifically, the application of a combination of VPH and Cu-VPH improved the yield of *O. basilicum* by regulating the photosynthetic efficiency and carboxylation capacity of the plants.

In summary, the research published broadly covers different aspects of microbial, extracts of various seaweeds and protein hydrolysate-derived products broadly falling in the biostimulant registration category, in improving plant growth, stress tolerance, and nutrient-use-efficiency. These collated articles provide a holistic insight into understanding the modes of actions of various types of biostimulants in plant development and nutrient-use-efficiency. These findings will lead to the development of next-generation biostimulants for sustainable agriculture. In future, it is expected that next-generation products will be synergistic formulations of multiple types of biostimulants, such as seaweed extracts with additions of protein extracts, humates, and microbial metabolites.

## Author contributions

SB: Conceptualization, Project administration, Writing – original draft. PS: Conceptualization, Project administration, Supervision, Writing – original draft, Writing – review & editing. BP: Writing – original draft, Writing – review & editing. AC: Writing – original draft, Writing – review & editing. NN: Writing – original draft, Writing – review & editing.
